# Fast and Flexible Movable Vision Measurement for the Surface of a Large-Sized Object

**DOI:** 10.3390/s150304643

**Published:** 2015-02-25

**Authors:** Zhen Liu, Xiaojing Li, Fengjiao Li, Xinguo Wei, Guanjun Zhang

**Affiliations:** Ministry of Education Key Laboratory of Precision Opto-Mechatronics Technology, Beihang University, No.37 Xueyuan Rd., Haidian District, Beijing 100191, China; E-Mails: liuzhen008@buaa.edu.cn (Z.L.); xjli_author@163.com (X.L.); chichi008@aspe.buaa.edu.cn (F.L.); g.j.zhang@hotmail.com (G.Z.)

**Keywords:** machine vision, binocular vision sensor, planar target

## Abstract

The presented movable vision measurement for the three-dimensional (3D) surface of a large-sized object has the advantages of system simplicity, low cost, and high accuracy. Aiming at addressing the problems of existing movable vision measurement methods, a more suitable method for large-sized products on industrial sites is introduced in this paper. A raster binocular vision sensor and a wide-field camera are combined to form a 3D scanning sensor. During measurement, several planar targets are placed around the object to be measured. With the planar target as an intermediary, the local 3D data measured by the scanning sensor are integrated into the global coordinate system. The effectiveness of the proposed method is verified through physical experiments.

## 1. Introduction

In the process of manufacture and assembly of large-sized objects the use of sensor feedback to guide the processing and assembly by the rapid online measurement of large-sized surface morphologies can significantly improve the machine efficiency and quality of the resulting parts. With advances in computer technology, image processing, and pattern-recognition technologies, vision measurement technology has rapidly developed. Vision measurement systems have gradually become the most important means of three-dimensional (3D) surface topography measurement for large-sized objects [1,2,3,4,5]. Currently, the main characteristic of 3D surface topography measurements for large-sized objects is that the measuring position is essentially fixed because in the industrial field each batch of large-sized product does not change significantly in shape. However, the fast-paced production and the small measuring space require that the measurement system be highly accurate, its speed rapid, and the structures be simple and flexible. However, most existing vision measurement systems cannot meet the needs of fast-paced on-site production. Thus, research on fast and high-precision 3D shape measurement of large-sized objects is important in the industrial field.

At present, 3D shape measurement of objects basically uses three methods: structured light vision measurement, Fourier profilometry, and phase profilometry. Structured light vision measurement includes the multi-line structured light method and the encoding structured light method. Light strip matching is more difficult, so the multi-line structured light method [6,7] is usually applied in object geometric measurement. Meanwhile, the coded structured light method [8,9,10] is an effective means of obtaining dense 3D point clouds of object 3D surface morphology; the method operates on a simple principle and has a high degree of automation, so it is the most commonly used among the 3D shape measurement methods. The biggest advantage of Fourier transform profilometry [11,12,13] is that by using only one image, it can achieve 3D object surface topography measurement. Therefore, it is suitable for dynamic 3D measurements. Its disadvantages are its long operation time and low automated performance, so it is not suitable for industrial measurements. Phase measurement profilometry [14,15,16] has high accuracy and is currently the most frequently used 3D shape measurement method, however, the algorithm is more complex and the phase unwrapping problem exists.

Single-vision sensors cannot achieve overall large-sized object 3D surface topography measurement because of the occlusions. The usual method is to divide the area to be measured into a plurality of sub-regions, and all the sub-region 3D data are integrated into the global coordinate system to obtain a 3D morphology of the entire surface of the object. Depending on the different overall unified approaches, the surface 3D morphology vision measurement of large-sized objects can be divided into two categories: movable single-vision sensor measurement and fixed multiple vision sensor measurement.

Movable single-vision sensor measurement method measures the 3D morphology of the entire surface of a large-sized object using a movable single-vision sensor. It uses simple equipment and is low cost. This method affixes labels on the object or uses a planar target to integrate the subregion 3D data into the global coordinate system. A typical method using adhesive markers is the ATOS movable 3D optical measurement system developed by the GOM Company. However, many of the objects to be measured (e.g., soft objects, liquids, or high-precision mechanical components) cannot be labeled. Meanwhile, this method has a long adhesive marking time and the marker is easily deformed. By contrast, using the planar target method [17], errors caused in the single movable measurement can easily accumulate. In addition, the planar target needs to be placed before the measured object in this method, the measuring time is long, and the operation is complex.

Fixed multiple-vision sensor measurement [18,19,20] needs to employ more on-site vision sensors and complete the global calibration of multiple vision sensors. Based on the global calibration results, data obtained by each vision sensor are united into the global coordinate system. Currently, typical measurement systems using fixed multiple-vision sensors include those of the American company Perceptron, with its auto-body geometry detection system, and Italian company MERMEC, with its online trial of full profile measurement systems. The principle of the method is simple, but the systems are complex and on-site calibration is difficult. After the measurement system is moved or the measured objects are changed, the measurement system needs to be reconfigured and recalibrated. At present, the method is often used in the geometry size measurement of the mass-produced large-sized products in the industrial field, but it is not suitable for large-sized and complex 3D surface reconstruction.

Based on the above analysis, compared with the fixed multiple-vision sensors measurement, the movable single-vision sensor measurement method seems more suitable for 3D surface topography measurement of the large-sized object. To achieve rapid measurement of the 3D surface topography of large-sized object, particularly mass-produced large-sized objects in the industrial field, the method proposed herein combines a raster binocular vision sensor with a wide-field camera to form a 3D scanning sensor. Multiple plane targets arranged in the surroundings of the measured object are used as intermediaries and the local 3D data obtained from the 3D scanning sensor are integrated into the global coordinate system. The remainder of the paper is organized as follows: Section 2 describes the structure and the mathematical model of the 3D scanning sensor, Section 3 provides a detailed description of the basic principles of the algorithm, and Section 4 verifies the effectiveness of the proposed algorithm through experiments.

## 2. System Measurement Principle

**Figure 1:**
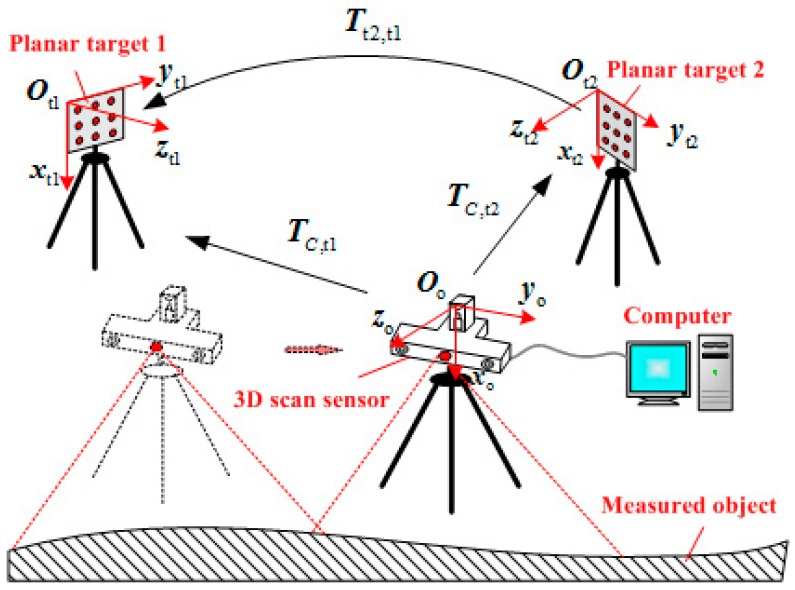
The structural schematic of the measurement system.

The structural schematic of the measurement system is shown in Figure 1. The coordinate systems of planar targets 1 and 2 are $O_{t1}x_{t1}y_{t1}z_{t1}$ and $O_{t2}x_{t2}y_{t2}z_{t2}$, respectively. $O_{\text{o}}x_{\text{o}}y_{\text{o}}z_{\text{o}}$ is the 3D scanning sensor coordinate system. The coordinate system of planar target 1 $O_{t1}x_{t1}y_{t1}z_{t1}$ is selected as the global coordinate system $O_{G}x_{G}y_{G}z_{G}$. The 3D scanning sensor is placed in front of the measured object to ensure that the wide-field camera can “see” the target plane. $T_{C,t1}$ and $T_{C,t2}$ are the transformation matrices from the 3D scanning sensor coordinate system $O_{\text{o}}x_{\text{o}}y_{\text{o}}z_{\text{o}}$ to the coordinate systems of plane targets 1 and 2, respectively. $T_{t2,t1}$ is the transformation matrix from the plane target 2 to plane target 1.

The proposed system includes a 3D scanning sensor, multiple planar targets, a high-speed image acquisition system, a computer, measurement software, and the corresponding mechanical structure. The basic principle of the measurement system is as follows: first, multiple planar targets are arranged around the measured object. Second, the raster binocular stereo vision sensor of the 3D scanning sensor measures the local 3D surface of the object, and the wide-field camera of the 3D scanning sensor measures the planar target. Finally, these planar targets function as the mediators to integrate all local 3D data measured by the 3D scanning sensor into the global coordinate system $O_{G}x_{G}y_{G}z_{G}$.

### 2.1. 3D Scanning Sensor

**Figure 2:**
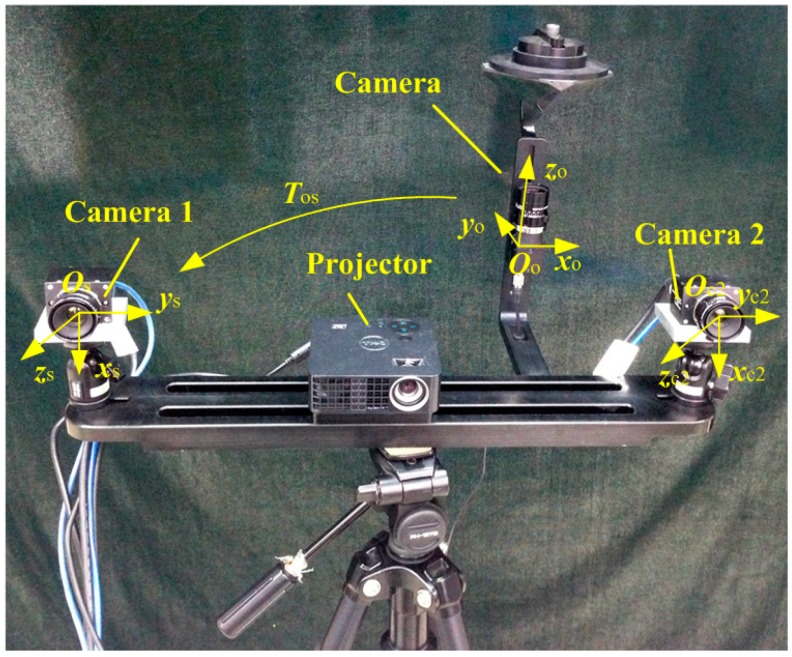
Structure of the 3D scanning sensor.

**Figure 3:**
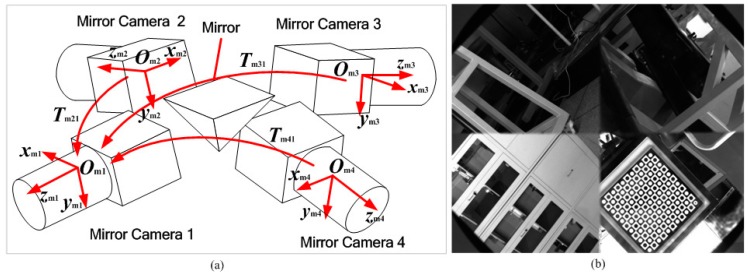
(**a**) Schematic image of the wide-field camera; (**b**) Image captured by the wide-field camera.

As shown in Figure 2, the 3D scanning sensor includes a raster binocular stereo vision sensor and a wide-field camera. The raster binocular stereo vision sensor consists of two cameras and a projector. The wide-field camera is a combination of a high-resolution camera and a four-sided mirror. The wide-field camera also can be considered as a four-mirror camera to achieve multi-angle measurement. The 3D scanning sensor coordinate system $O_{\text{o}}x_{\text{o}}y_{\text{o}}z_{\text{o}}$ is established under the wide-field camera coordinate system $O_{\text{l}}x_{\text{l}}y_{\text{l}}z_{\text{l}}$. The raster binocular stereo vision sensor coordinate system $O_{\text{s}}x_{\text{s}}y_{\text{s}}z_{\text{s}}$ is established under the left camera coordinate system $O_{c1}x_{c1}y_{c1}z_{c1}$ of the raster binocular vision sensor and $T_{\mathrm{os}}$ is the transformation matrix from $O_{\text{o}}x_{\text{o}}y_{\text{o}}z_{\text{o}}$ to $O_{\text{s}}x_{\text{s}}y_{\text{s}}z_{\text{s}}$. Schematic images of the wide-field camera and the image captured by the wide-field camera are shown in Figure 3a,b, respectively.

Compared with the curved mirror in current panoramic cameras, the model of the wide-field camera with a four-surface mirror is simpler and its measurement accuracy is higher. However, the field of views of the wide-field camera has a blind area. The size and measuring location of mass-produced large-sized products are basically fixed in industrial production sites, so each movement position of the 3D scanning sensor can be determined in advance, and the planar targets used in the proposed method can optimize those positions based on each moving point of the 3D scanning sensor. Therefore, compared with using a panoramic camera with curved mirrors, the wide-field camera with a four-surface mirror is suitable for industrial production. It is also the main reason why flat mirrors are used in the proposed method instead of curved mirrors.

As shown in Figure 3a, $\begin{array}{cc} O_{\text{m}i}x_{\text{m}i}y_{\text{m}i}z_{\text{m}i} & \left(i=1,2,3,4\right) \end{array}$ is the coordinate system of the four mirror cameras of the wide-field camera, respectively. $T_{\text{m}21},T_{\text{m}31},T_{\text{m}41}$ are the transformation matrixes from the coordinate system of mirror cameras 2, 3, and 4 to that of mirror camera 1, respectively. The coordinate system $O_{\text{l}}x_{\text{l}}y_{\text{l}}z_{\text{l}}$ of the wide-field camera is established based on the coordinate system $O_{\text{m}1}x_{\text{m}1}y_{\text{m}1}z_{\text{m}1}$ of mirror camera 1. According to Equation (1), the 3D coordinates $P_{\text{o}}=[x_{\text{o}},y_{\text{o}},z_{\text{o}},1]$ of *P* under the coordinate system $O_{\text{o}}x_{\text{o}}y_{\text{o}}z_{\text{o}}$ can be obtained under the coordinate system of the 3D scanning sensor:

(1) $$P_{\text{o}}=T_{\mathrm{os}}^{-1}P_{\text{s}}$$

where $P_{\text{s}}$ is the 3D coordinates of P under the coordinate system $O_{\text{s}}x_{\text{s}}y_{\text{s}}z_{\text{s}}$. $T_{\mathrm{os}}$ can be obtained by calibration before measuring [21].

### 2.2. Light Strip Image Center Extraction and Coding

#### Light Strip Coding

**Figure 4:**
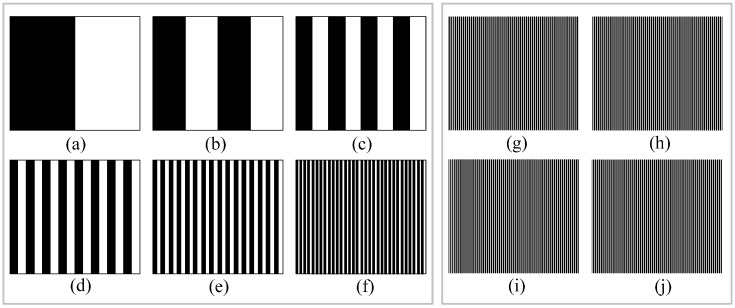
All projected images.

The proposed method uses the existing binary-coded method [22] to achieve the match with the left and right cameras’ light strips. The projector is first arranged according to Figure 4a–f to cast six black and white images. Supposing that the black is defined as 0 and the white is 1. The coding index of the first black light bar region in the left side of the 64 black and white light bar region is 000000 in Figure 4f. From left to right, the successive light bar coding is 000001, 000010, 000011, and so on.

**Figure 5:**
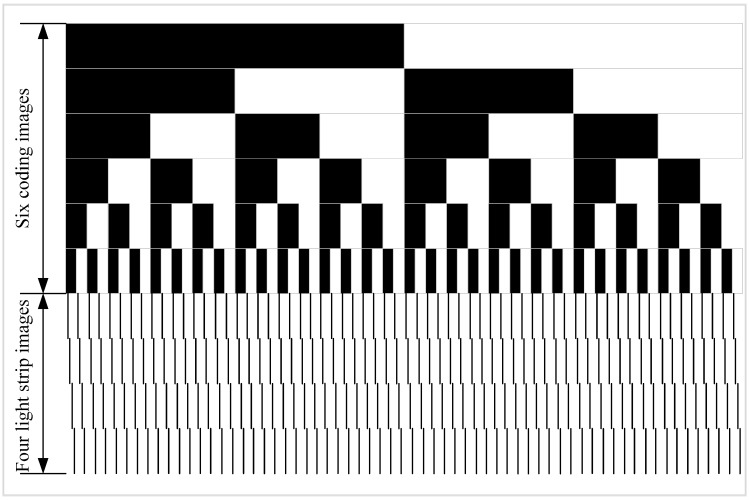
Line distribution diagram of all projected images.

As shown in Figure 4a–f, after the projection of six black and white images, 64 black and white light bar regions can be formed, and they have been encoded. Four light strip images are constructed, and each image has 64 vertical light strips, as shown in Figure 4g–j. In each graph of Figure 4g–j, 64 light strips are in 64 black and white coding light bar regions formed in Figure 4a–f. According to the four light strip images above, 64 × 4 = 256 light strips can be obtained. The distribution schematic diagram of all projected images is shown in Figure 5. At the same time, the number of light strip images can be increased or decreased based on need; 64 × 2 = 128 light strips can be obtained by projecting two images, 64 × 6 = 384 light strips can be obtained by projecting six images, and so on.

**Figure 6:**
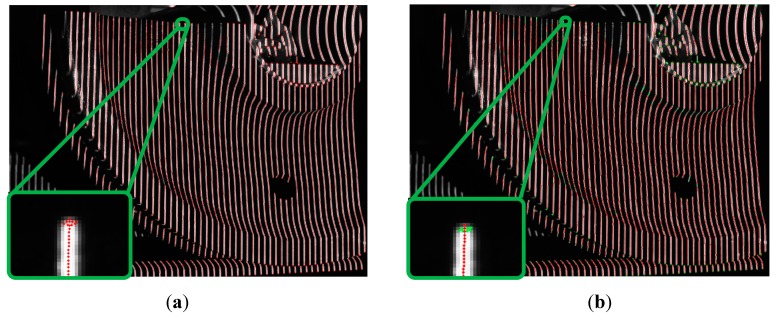
Light stripe extraction results. (**a**) Extraction result of the sub-pixel coordinates of the light stripe center; (**b**) Extraction result of the light stripe center after linking.

In Figure 4a–f, the black and white light bar region is only used for constructing the coding region to identify 64 light strips of each light strip image in Figure 4g–j. In the four light strip images shown in Figure 4g–j, the Steger [23] algorithm is used to extract the light strip center point of the light strip image in this paper. First, the Hessian matrix is used to determine the pixel-level coordinate and the normal direction of the light strip center Then, the sub-pixel level coordinate of the light strip center is obtained by solving the extreme points in the normal direction, as shown in Figure 6a. Finally, a link constraint method is used to remove the wrong center of the light bar and to link the correct light strip centers together to form a plurality of segments, as shown in Figure 6b.

### 2.3. Partial 3D Reconstruction

Section 2.2 shows that each light strip in the projected image corresponds to a unique code index. The light strips captured by the left and right cameras of the raster binocular stereo vision sensor can be matched according to the code index. The corresponding points of the light strip captured by two cameras can be obtained according to the epipolar constraints. Finally, the corresponding points are substituted into the raster binocular stereo vision model to calculate the 3D coordinates of the corresponding points.

**Figure 7:**
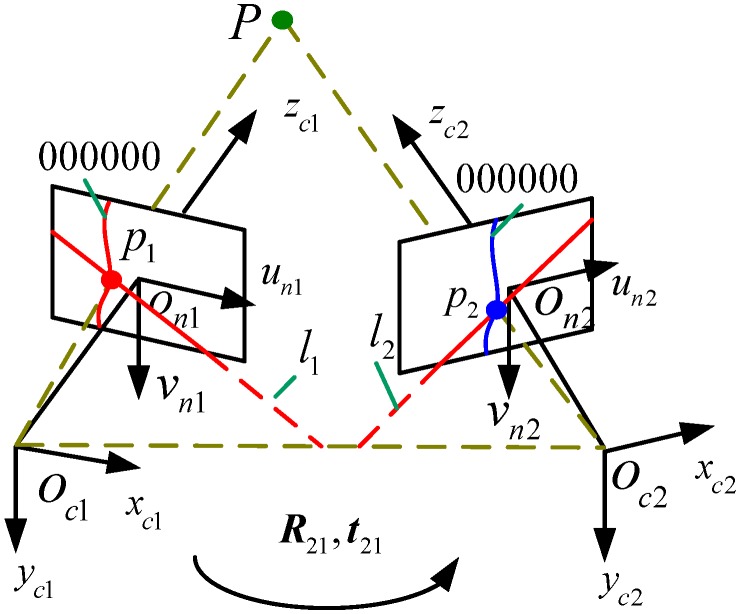
Schematic of the grating binocular vision sensor model.

The schematic of the grating binocular vision sensor model is shown in Figure 7. The left camera’s coordinate system is $O_{c1}x_{c1}y_{c1}z_{c1}$ and the right camera’s coordinate system is $O_{c2}x_{c2}y_{c2}z_{c2}$. The transformation matrix from $O_{c1}x_{c1}y_{c1}z_{c1}$ to $O_{c2}x_{c2}y_{c2}z_{c2}$ is $T_{21}=\left[\begin{array}{cc} R_{21} & t_{21}\\ 0 & 1 \end{array}\right]$ (where $R_{21}\;\text{and}\;t_{21}$ are the rotation matrix and translation vectors, respectively).

Without loss of generality, the raster binocular stereo vision sensor coordinate system $O_{\text{s}}x_{\text{s}}y_{\text{s}}z_{\text{s}}$ is built under $O_{c1}x_{c1}y_{c1}z_{c1}$. $p_{1}={\left[u_{1},v_{1},1\right]}^{T}$ and $p_{2}={\left[u_{2},v_{2},1\right]}^{T}$ are undistorted homogeneous image coordinates of the light strip point P at the left and right camera coordinate system (obtained from the distorted image of homogeneous coordinates through lens distortion correction [24]). $l_{1}$ is the pole line of $p_{2}$ in the left camera and $l_{2}$ is the pole line of $p_{1}$ in the right camera. The 3D coordinates $P_{\text{s}}={\left[x_{\text{s}},y_{\text{s}},z_{\text{s}},1\right]}^{T}$ of P under $O_{\text{s}}x_{\text{s}}y_{\text{s}}z_{\text{s}}$ can be solved by the binocular stereo vision model, as shown in Equation (2):

(2) $$\left\{ \begin{array}{l} \rho_{1}p_{1}=K_{1}\left[\begin{array}{cc} I & 0 \end{array}\right]P_{\text{s}}\\ \rho_{2}p_{2}=K_{2}\left[\begin{array}{cc} R_{21} & t_{21} \end{array}\right]P_{\text{s}} \end{array}\right.$$

where $K_{1}$ and $K_{2}$ are intrinsic parameters of the left and right cameras, respectively.

The light strip matching of the left and right cameras can be achieved by light strip coding. The epipolar constraints are added to achieve the corresponding light strip center points of the left and right cameras. The corresponding points are substituted into Equation (4) to calculate their 3D coordinates.

### 2.4. Global Unity of Partial 3D Data

The partial 3D reconstruction process of the 3D scanning sensor is introduced in Section 2.3. However, limited by the vision sensor’s field of view, the partial 3D reconstruction of the 3D scanning sensor can only measure the local 3D data of large-sized objects. To achieve the overall 3D reconstruction of large-sized objects, all local 3D data need to be integrated into the global coordinate system.

The planar target 1 coordinate $O_{t1}x_{t1}y_{t1}z_{t1}$ is selected as the global coordinate system $O_{\text{G}}x_{\text{G}}y_{\text{G}}z_{\text{G}}$. $P_{\text{O}}$ is the 3D coordinate of the light strip center point *P* measured by the raster binocular vision sensor in the 3D scanning sensor coordinate system $O_{\text{O}}x_{\text{O}}y_{\text{O}}z_{\text{O}}$ and $P_{\text{G}}$ is the 3D coordinate of *P* in the global coordinate system $O_{\text{G}}x_{\text{G}}y_{\text{G}}z_{\text{G}}$. As shown in Figure 1, the wide-field camera of the 3D scanning sensor can “see” two plane targets placed around the large-sized object to calculate $T_{C,t1}$ and $T_{C,t2}$, then calculate $T_{t2,t1}$. The local 3D coordinates measured by 3D scanning sensor can be integrated into the global coordinate system using Equation (3):

(3) $$P_{\text{G}}=T_{C,t1}P_{\text{O}}$$

The wide-field camera may not “see” planar target 1, but it can “see” plane target 2. Then, the local 3D data can be integrated into the global coordinate system using Equation (4):

(4) $$P_{\text{G}}=T_{t2,t1}T_{C,t2}P_{\text{O}}$$

To improve system efficiency and flexibility, multiple plane targets can be arranged around the large-sized object on the measurement site.

## 3. Physical Experiments

**Figure 8:**
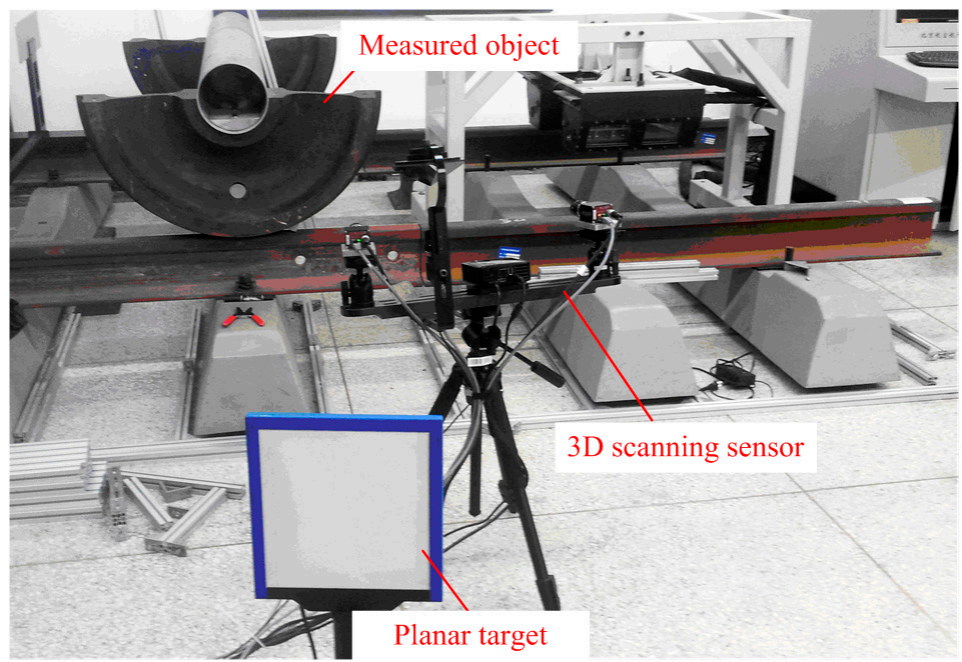
Layout of the physical experiments.

The setup of the physical experiment is shown in Figure 8. The raster binocular stereo vision sensor of the 3D scanning sensor consists of two cameras (AVTGC1380H with 17 mm lenses and a resolution of 1360 × 1024 and a field of view of 500 mm × 380 mm × 400 mm, Allied Vision Technologies, Stadtroda, Germany) and one projector (Dell M110 with a resolution of 1360 × 768, Dell, Round Rock, TX, USA). The wide-field camera of the 3D scanning sensor consists of one camera (Pointgray with 12 mm lenses and a resolution of 2448 × 2048, Point Grey Research, Richmond, Canada) and one mirror with four surfaces. The characteristic point of the planar target is unified as 10 × 10, with a machining accuracy of 5 μm.

### 3.1. System Calibration Results

First, the raster binocular stereo vision and the intrinsic parameters of the wide-field camera are calibrated based on [24,25]. Then, the transformation matrix $T_{m21}$, $T_{m31}$, $T_{m31}$ and $T_{\mathrm{os}}$ are calibrated by [21]. The planar target used for calibration is the same as the planar target for the measurement system.

All calibration results of 3D scanning sensor are shown as follows:

(1) Binocular stereo vision sensor

Intrinsic parameters of left camera: *fx* = 2744.3; *fy* = 2745.9; γ = 0; *u*_0_ = 750.2; *v*_0_ = 480.6; *k*_1_ = −0.14; *k*_2_ = −0.51.

Intrinsic parameters of right camera: *fx* = 2754.6; *fy* = 2753.5; γ = 0; *u*_0_ = 709.7; *v*_0_ = 538.4; *k*_1_ = −0.15; *k*_2_ = 0.02.

***T***_21_: ***r***_21_ = [0.0387, 0.416, 0.00024]; ***t***_21_ = [−489.501, 13.515, 90.88667].

The uncertainty of intrinsic parameters of left camera: $u_{f_{x}}=1.75$; $u_{f_{y}}=1.89$; $u_{u_{0}}=3.67$; $u_{v_{0}}=2.18$; $u_{k_{1}}=5.0\times 10^{-3}$; $u_{k_{2}}=6.29\times 10^{-2}$.

The uncertainty of intrinsic parameters of right camera: $u_{f_{x}}=1.70$; $u_{f_{y}}=1.74$; $u_{u_{0}}=3.43$; $u_{v_{0}}=2.52$; $u_{k_{1}}=5.91\times 10^{-3}$; $u_{k_{2}}=7.51\times 10^{-2}$.

A planar target is placed before the binocular stereo vision sensor at two positions, which measures the distance of character points of the target. Compared the real distance and the measurement distance, the RMS error is 0.09 mm. The binocular stereo vision sensor measure the character points of planar target 100 times, the deviation error is 0.03 mm.

(2) Wide-field camera

Intrinsic parameters: *fx* = 3705.8; *fy* = 3706.5; *γ* = 0; *u*_0_ = 1222.5; *v*_0_ = 997.4; *k*_1_ = −0.14; *k*_2_ = 0.26.

***T***_m21_: ***r***_m21_ = [−0.036, −1.528, −0.798]; ***t***_m21_ = [85.327, −55.294, 94.977].

***T***_os_: ***r***_os_ = [1.413, 1.002, −1.257]; ***t***_os_ = [97.625, −103.644, 261.717].

To verify the effectiveness of the proposed method, the following experiments were conducted to evaluate the measurement accuracy. The following subsection is a detailed description of the procedures and results.

**Figure 9:**
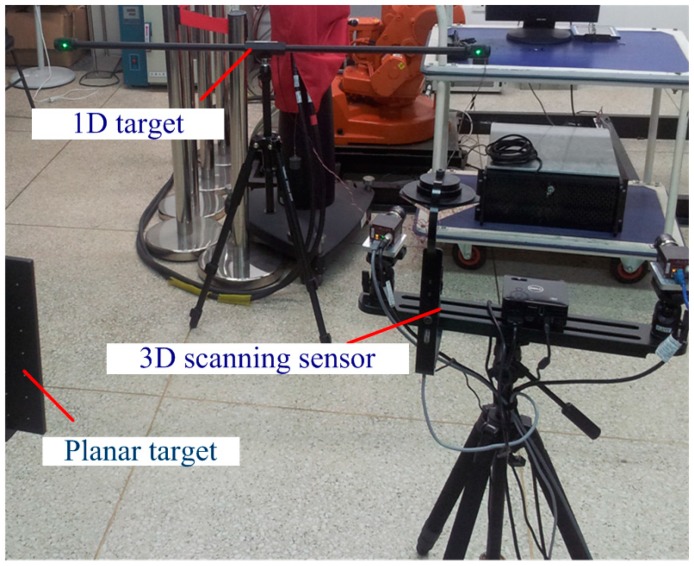
Schematic of global measurement precision evaluation.

A self-designed method is used to evaluate the global measurement precision. The specific experimental procedure is as follows: a one-dimensional (1D) target with two characteristic points (the distance between the two points is 1234.15 mm, with a precision of 0.01 mm) is placed before the 3D scanning sensor, which measures the characteristic point of the 1D target, as shown in Figure 9. Firstly, the 3D scanning sensor measures the left characteristic point of the 1D target at the first position, then it measures the right characteristic point of the 1D target at the second position. Finally, all characteristic points of 1D target measured by the scanning sensor at two positions are integrated into the global coordinate system by the planar target. The above progress is repeated eight times. The distance between the two points is calculated as the measurement distance (*d*m). The real distance between the two points of 1D target is the ideal distance (*d*t = 1234.15 mm). The deviation (Δ*d*) between *d*_m_ and *d*_t_ and the RMS error are calculated to evaluate the global accuracy of the proposed method.

**Figure 10:**
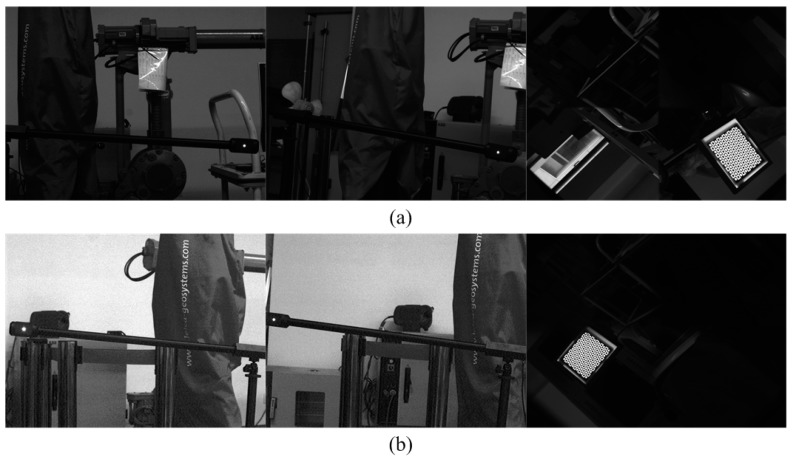
Images captured by two sensors for global precision calibration.

The images captured by the wide-field camera and the two cameras of the raster binocular stereo vision sensor at the first position are shown in Figure 10a. The images captured by the wide-field camera and the two cameras of the raster binocular stereo vision sensor at the second position are shown in Figure 10b. The distances between the two points of the 1D target by the 3D scanning sensor at eight positions and the RMS error are listed in Table 1. The result shows the global measurement accuracy of the proposed method can reach 0.14 mm.

**Table 1:** Evaluation of the global measurement accuracy (mm).

	Left Point			Right Point			*d*t (mm)	*d*m (mm)	Δ*d* (mm)
	*x* (mm)	*y* (mm)	*z* (mm)	*x* (mm)	*y* (mm)	*z* (mm)			
1	−38.93	69.70	944.18	1136.60	10.56	1315.73	1234.15	1234.27	−0.12
2	−58.28	−0.67	942.06	1145.09	34.81	1213.41	1234.15	1234.06	0.09
3	−207.67	163.54	1177.13	1002.70	13.97	988.70	1234.15	1234.05	0.10
4	−253.61	44.32	1147.33	915.61	294.11	1452.90	1234.15	1234.04	0.11
5	109.48	102.07	852.49	1265.76	31.61	1278.49	1234.15	1234.27	−0.12
6	−214.85	36.15	1012.34	1011.10	162.10	942.91	1234.15	1233.36	−0.21
7	55.09	16.89	1160.02	1160.73	67.45	613.74	1234.15	1234.27	−0.12
8	58.98	1.35	838.59	1191.43	−109.66	1316.00	1234.15	1233.97	0.18
							RMS error		0.14

### 3.2. Real Data Measurement Experiment

To verify the effectiveness of the proposed method, the following real data measurement experiment was designed. In the experiment, the 3D scanning sensor is moved twice to measure the 3D morphology of the two parts of the train wheels.

**Figure 11:**
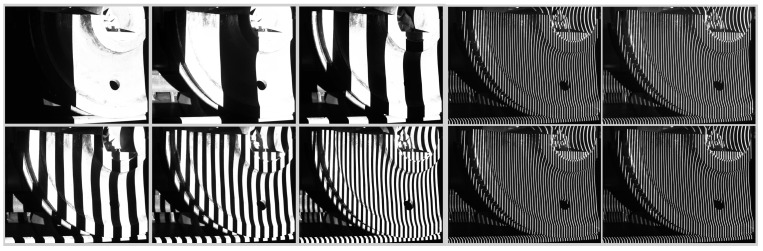
Coded image and light strip image captured by 3D scanning sensor at the first position.

**Figure 12:**
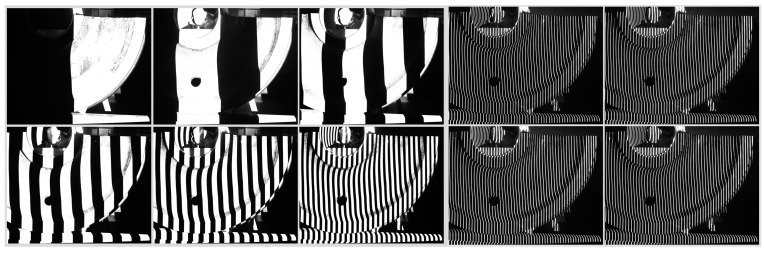
Coded image and light strip image captured by 3D scanning sensor at the second position.

The wide-field camera of the 3D scanning sensor is used to measure the arranged planar target. With the plane target as an intermediary, the local 3D data from two measurements is united to the global coordinate system. The coded images and the light strip images are shown in Figure 11 and Figure 12, respectively.

**Figure 13:**
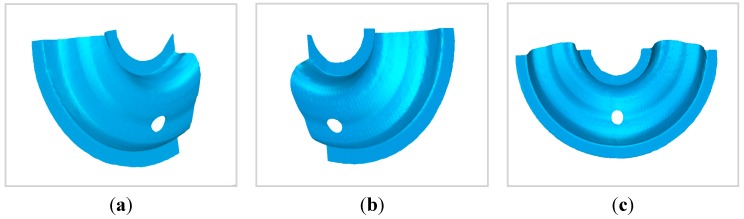
(**a**) 3D morphology of the object measured by the 3D scanning sensor at the first position; (**b**) 3D morphology of the object measured by the 3D scanning sensor at the second position; (**c**) united 3D morphology of the object measured by the 3D scanning sensor at two positions.

The 3D morphology of the object measured by the 3D scanning sensor at the first position is shown in Figure 13a. The 3D morphology of the object measured by the 3D scanning sensor at the second position is shown in Figure 13b. The united 3D morphology of the object measured by the 3D scanning sensor at two positions is shown in Figure 13c.

**Figure 14:**
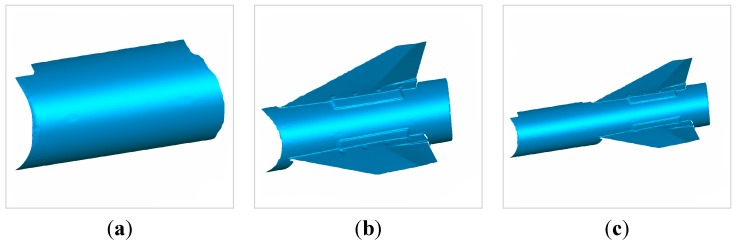
(**a**) 3D morphology of the missile model measured by the 3D scanning sensor at the first position; (**b**) 3D morphology of the missile model measured by the 3D scanning sensor at the second position; (**c**) united 3D morphology of the missile model measured by the 3D scanning sensor at two positions.

In order to further validate the effectiveness of the proposed algorithm, we measured the missile model, and their 3D morphology are shown in Figure 14.

## 4. Conclusions

Given that existing movable vision measurement methods for the 3D surface of large-sized objects have some problems, such as long operation times, low efficiency, and unsuitability for soft surfaces, a fast and high-precision movable vision measurement method for 3D surface of large-sized objects is introduced in this paper.

Compared with the existing measurement methods, the proposed method combines a raster binocular vision sensor with a wide-field camera to form a scanning sensor, and it is not necessary to past marks on the object’s surface in front of the object. Meanwhile, the proposed method realizes the synchronous measurement of partial 3D measurement and local 3D data integrating, which is not needed to move target repeatedly in front of the object and the 3D scanning sensor, so it greatly improving the measurement efficiency. Physical experiment confirms that when the size of 1D target is about 1.2 m, the accuracy of the proposed method could reach 0.14 mm. Meanwhile, the proposed method is s of high flexibility and efficiency.

Since the size and measuring location of mass-produced large-sized products are basically fixed, so each moved position of the 3D scanning sensor can be determined in advance, and those positions of the planar targets used in the proposed method can be optimized based on each moving point of the 3D scanning sensor. Thus, the proposed method is especially suitable for the measurement for the mass-produced large-sized products in the industrial site.

## References

[1] Chen F., Brown G.M., Song M. (2000). Overview of three-dimensional shape measurement using optical methods. Opt. Eng..

[2] Malamas E.N., Petrakis E.G.M., Zervakis M., Petit L., Legat J.D. (2003). A survey on industrial vision systems, applications and tools. Image Vis. Comput..

[3] Kovac I. Flexible inspection system in the body-in white manufacturing. Proceedings of the International Workshop on Robot Sensing, 2004 (ROSE 2004).

[4] Okamoto A., Wasa Y., Kagawa Y. (2007). Development of shape measurement system for hot large forgings. Kobe Steel Eng. Rep..

[5] Furferi R., Governi L., Volpe Y., Carfagni M. (2013). Design and assessment of a machine vision system for automatic vehicle wheel alignment. Int. J. Adv. Robot. Syst..

[6] Zeng L., Hao Q., Kawachi K. (2000). A scanning projected line method for measuring a beating bumblebee Wing. Opt. Commun..

[7] Jang W., Je C., Seo Y., Lee S.W. (2013). Structured-light stereo: Comparative analysis and integration of structured-light and active stereo for measuring dynamic shape. Opt. Lasers Eng..

[8] Morano R.A., Ozturk C., Conn R., Dubin S., Zietz S., Nissano J. (1998). Structured light using pseudorandom codes. IEEE Trans. Pattern Anal. Mach. Intell..

[9] Salvi J., Pagès J., Batlle J. (2004). Pattern codification strategies in structured light systems. Pattern Recognit..

[10] Koninckx T., Griesser A., van Gool L. Real-time range scanning of deformable surfaces by adaptively coded structured light. Proceedings of the International Conference on 3-D Digital Imaging and Modelling.

[11] Su X.Y., Chen W.J., Zhang Q.C., Chao Y.P. (2001). Dynamic 3-D shape measurement method based on FTP. Opt. Lasers Eng..

[12] Zappa E., Busca G. (2012). Static and dynamic features of Fourier transform profilometry: A review. Opt. Lasers Eng..

[13] Su X.Y., Zhou W.S., Bally G., Vukicevic D. (1992). Automated phased-measuring profilometry using defocused projection of a Ronchi grating. Opt. Commun..

[14] Quan C.G., Chen W., Tay C.J. (2010). Phase-retrieval techniques in fringe-projection profilometry. Opt. Lasers Eng..

[15] Huang P.S., Zhang C.P., Chiang F.P. (2003). High-speed 3-D shape measurement based on digital fringe projection. Opt. Eng..

[16] Zhang S. (2010). Recent progresses on real-time 3D shape measurement using digital fringe projection techniques. Opt. Lasers Eng..

[17] Sun J.H., Zhang G.J., Wei Z.Z., Zhou F.Q. (2006). Large 3D free surface measurement using a movable coded light-based stereo vision system. Sens. Actuators A Phys..

[18] Lu R.S., Li Y.F., Yu Q. (2001). On-line measurement of straightness of seamless steel pipe using machine vision technique. Sens. Actuators A Phys..

[19] Li Q., Ren S. (2012). A Real-Time Visual Inspection System for Discrete Surface Defects of Rail Heads. IEEE Trans. Instrum. Meas..

[20] Li Y., Li Y.F., Wang Q.L., Xu D., Tan M. (2010). Measurement and defect detection of the weld bead based on online vision inspection. IEEE Trans. Instrum. Meas..

[21] Liu Z., Zhang G.J., Wei Z.Z., Sun J.H. (2011). A global calibration method for multiple vision sensors based on multiple targets. Meas. Sci. Technol..

[22] Posdamer J.L., Altschuler M.D. (1982). Surface measurement by space-encoded projected beam systems Comput. Graph. Image Process.

[23] Steger C. (1998). An unbiased detector of curvilinear structures. IEEE Trans. Pattern Anal. Mach. Intell..

[24] Zhang Z.Y. (2000). A flexible new technique for camera calibration. IEEE Trans. Pattern Anal. Mach. Intell..

[25] Bouguet J.Y. Camera Calibration Toolbox for Matlab. http://www.vision.caltech.edu/bouguetj/calib_doc/.

